# Stem Cell Therapies in Kidney Diseases: Progress and Challenges

**DOI:** 10.3390/ijms20112790

**Published:** 2019-06-07

**Authors:** Cinzia Rota, Marina Morigi, Barbara Imberti

**Affiliations:** Istituto di Ricerche Farmacologiche Mario Negri IRCCS, Centro Anna Maria Astori, Science and Technology Park Kilometro Rosso, Via Stezzano 87, 24126 Bergamo, Italy; cinzia.rota@marionegri.it (C.R.); barbara.imberti@marionegri.it (B.I.)

**Keywords:** stem cells, kidney diseases

## Abstract

The prevalence of renal diseases is emerging as a public health problem. Despite major progress in supportive therapy, mortality rates among patients remain high. In an attempt to find innovative treatments to stimulate kidney regeneration, stem cell-based technology has been proposed as a potentially promising strategy. Here, we summarise the renoprotective potential of pluripotent and adult stem cell therapy in experimental models of acute and chronic kidney injury and we explore the different mechanisms at the basis of stem cell-induced kidney regeneration. Specifically, cell engraftment, incorporation into renal structures, or paracrine activities of embryonic or induced pluripotent stem cells as well as mesenchymal stem cells and renal precursors are analysed. We also discuss the relevance of stem cell secretome-derived bioproducts, including soluble factors and extracellular vesicles, and the option of using them as cell-free therapy to induce reparative processes. The translation of the experimental results into clinical trials is also addressed, highlighting the safety and feasibility of stem cell treatments in patients with kidney injury.

## 1. Introduction

The increasing incidence of kidney diseases raises considerable concerns regarding human health worldwide. The pressure to find new strategies to prevent or halt the progression, or resolve kidney disease, is driven by the limited availability of therapies.

The long path towards understanding how the kidney works and how it can be protected and healed began over two centuries ago [1].

Since then, great progress has been made, beginning with basic science in the mid-1800s, which furthered our knowledge of renal filtration and fluid maintenance, up until the breakthrough in the 1940s of the first attempt to create an artificial kidney [2]. The decades that followed were characterised by the first long-term successful human kidney transplantation from a living donor carried out by Dr Joseph Murray, who received the Nobel Prize in Medicine for this achievement [3,4]. This was a huge step forward but highlighted the need to better understand the immune system to prevent rejection [5]. Further significant improvements were pursued later in the 1980s, which were achieved by the introduction of HLA-DR matching and the use of immunosuppressive agents, such as cyclosporine.

**Figure 1:**
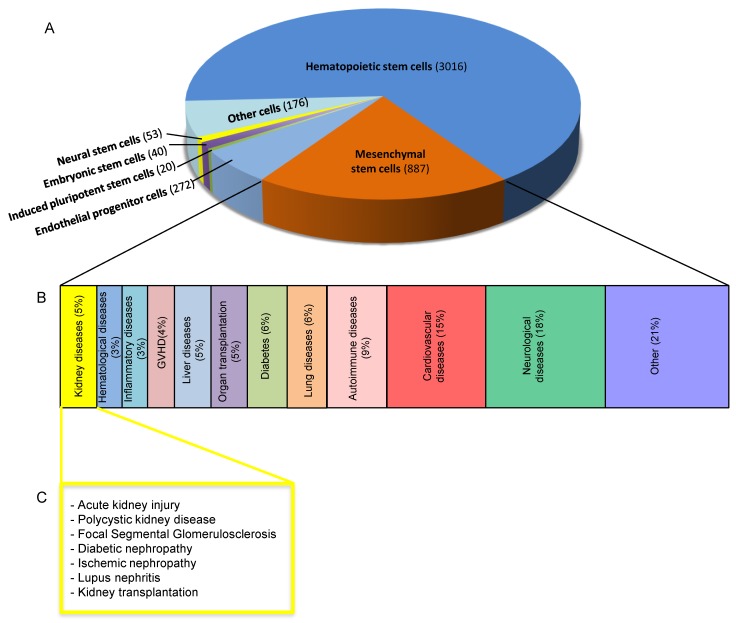
Clinical trials with cell-based therapy: embryonic and adult stem cells. (**A**) Pie chart showing the relative numbers of clinical trials using different types of stem cells as listed on the U.S. NIH website clinicaltrials.gov. (**B**) Percentage of MSC-based clinical trials classified by disease type. (**C**) MSC-based therapies in different kidney diseases.

The introduction in the 1960s of outpatient dialysis, a development that has had a great impact on patient quality of life, brought new hope to patients with chronic kidney diseases [6,7]. Since then, the new goal of delaying the progression of kidney disease was achieved through the discovery of drugs that inhibit the renin angiotensin aldosterone system (RAAS) [8]. It is not currently clear what the next necessary step is, but it seems to be in the direction of regenerative medicine. A number of studies in recent years have attempted to identify the underlying mechanisms of renal repair in order to explore the potential regenerative capacity of the kidneys. The main objective of this research has been to ascertain whether the regenerative capacity of adult kidneys can be supported by terminally differentiated cells, whether there are multipotent progenitor cells in the kidney and whether therapy with stem cells of extrarenal origin can contribute to renal repair, favouring or accelerating the regenerative process. Many papers have reported on the potential use of stem cells of different origins for treating many different pathologies, including kidney diseases. The efficacy, to varying extents, of using stem cells from different sources in models of acute kidney injury (AKI) and chronic kidney diseases (CKD) has been demonstrated experimentally. The field has generated great expectations, from the initial, widely dismissed results of stem cell integration into host renal tissue, to the paracrine hypothesis of stem cell-mediated repair. However, clinical translation of the experimental findings does not seem to be a realistic prospect in the near future, due principally to the lack of robustness of experimental data and to clinical studies that have been limited by modest outcomes and the small number of patients enrolled (Table 1, Figure 1). Before moving on to clinical applicability, many issues must still be resolved, including identifying the best cell types, the route and timing of administration, and the dose of cells necessary for different pathological conditions.

This review describes a wide range of pre-clinical reports on stem cell-based therapy in experimental AKI and CKD, summarizing current knowledge and discussing criticisms for moving to the clinic.

## 2. Acute Kidney Injury and Chronic Kidney Disease

Kidney disease is a severe health problem that affects over 10% of the global population and accounts for 5 to 10 million deaths annually mainly due to the progressive increase in obesity, diabetes, hypertension and cardiovascular disease [27]. Kidney diseases have been traditionally classified into two separated pathologies, acute kidney injury (AKI) and chronic kidney disease (CKD), even though a strict interconnection with regard to their aetiology has been recently highlighted [28]. AKI is a common clinical problem in critically ill patients with an incidence that varies between 15% and 50% [29] and it is associated with short- and long-term morbidity and mortality. AKI is characterised by the abrupt impairment of renal function, which can have pre-renal (i.e., hypoperfusion), intra-renal (i.e., nephrotoxic agents) or post-renal (obstruction of urinary tract) causes [30]. AKI is characterised by damage to the proximal tubular cells, which, unable to maintain adequate energy production, undergo apoptosis, detach and obstruct the lumen leading to increased intratubular pressure and “backleak” of filtrate contributing to dysfunction. In parallel, endothelial cell injury results in vascular rarefaction. AKI manifests clinically when a sudden increase in serum creatinine and nitrogen metabolites, reflecting a reduction in the glomerular filtration rate (GFR), or an abrupt reduction in urine output occurs [31]. As too many cases of AKI are diagnosed too late, major efforts are being made to discover biomarkers that can be used as early predictors of AKI. Some of these include mediators of inflammation (neutrophil gelatinase–associated lipocalin, IL-8), markers of changes in renal structure (kidney injury molecule-1), enzymes (alkaline phosphates, and alanine aminopeptidase) [32]. The management of AKI depends on its aetiology because the primary reversible cause of injury should be removed wherever possible. Nephrotoxic drugs should be avoided and fluid resuscitation and diuretic administration should be used to treat volume overload, even when they do not facilitate AKI recovery or reduce mortality [33]. Renal replacement therapy can also be required as a life-sustaining support strategy. Major efforts are being made to identify new targets for managing AKI, including molecules that act on hemodynamics, inflammation and oxidative states, cellular metabolism and mitochondrial function [34].

Clinical and experimental investigations have found an interconnected link between AKI and the development of CKD [35,36,37,38,39,40]. CKD can also be triggered by different risk factors, including cardiovascular pathologies, diabetes, nephrectomy, toxic insults, etc. The prevalence of CKD is rising worldwide, affecting about 18% of the global population [27]. CKD is a syndrome characterised by persistent changes to the kidney structure and function, which decreases progressively and is associated with nephron loss, glomerular hypertrophy, podocyte detachment and the formation of sclerotic lesions. Moreover, the atrophy of nephrons is accompanied by immune cell infiltration and interstitial fibrosis [41].

There are few therapeutic options for restoring a kidney affected by chronic disease [27] and the disease is often diagnosed too late because the initial stages can be asymptomatic. Severely damaged kidneys may progress to kidney fibrosis and end-stage renal disease (ESRD). ESRD requires renal replacement therapy that aims to substitute critical kidney function and consists of dialysis or kidney transplantation. As hypertension is recognised as one of the causes of CKD, the therapy for CKD patients is currently based on the use of drugs for blood pressure control and for inhibition of the renin-angiotensin aldosterone system, which plays a central role in controlling blood pressure. Only when treatment is started early, can the progression to ESRD be retarded. Therefore, new approaches to preventing and/or delaying the progression of CKD are urgently needed.

## 3. Stem Cells for Cell-Based Therapy

Great interest has been generated in the use of stem cells as a therapeutic option for regenerating damaged tissues and organs. Cell replacement therapy using stem cells would be based on generating a large number of cells in vitro. One of the most important features of stem cells is their capacity to divide and produce more stem cells (self-renewal potency and clonogenicity) or more differentiated precursors (asymmetric division), which is associated with their potential to differentiate into different specialised cell types (plasticity) [42]. However, not all stem cell types possess the same differentiative and therapeutic potential. They can be divided into two main categories based on potency: embryonic stem cells (ESCs) and adult stem cells.

ESCs are derived from the inner cell mass of the embryo at the blastocyst stage. They are self-renewing, clonogenic and pluripotent, meaning they are able to undergo lineage commitment into the three different embryonic derivatives: the ectoderm, mesoderm and endoderm [43]. These give rise to all cell types in the body. Several ESC lines have been derived from human embryos [44], but this has not been without ethical controversies. When human ESCs (hESCs) were successfully derived from single blastomeres without destroying the embryos [45], scientists thought they had found a strategy that allowed them to overcome moral concerns and accelerate hESC derivation. However, the use of this technique did not really take hold for these purposes and legislative issues continued to be a main impediment for research in this field. In spite of these difficulties, several different cell types have been derived from ESC differentiation, including neural cells [46], haematopoietic cells [47], cardiomyocytes [48] and pancreatic ß-cells [49], mainly for disease modelling purposes. Their use as cell therapy is limited by safety concerns. Indeed, ESCs have an unlimited cell renewal capacity, which is the main advantage of using them to obtain rapid and adequate cell expansion in vitro; however, they also carry the risk of uncontrolled growth and teratoma formation if they are not adequately differentiated. Another limit to their use is their immunogenicity, which induces rejection by the recipient’s immune system. In spite of all of these hurdles, clinical trials involving their use in spinal cord injury, macular degeneration of the retina, heart repair, and diabetes [50] have been initiated.

Over a decade ago, a novel reprogramming technology led to the generation of induced pluripotent stem cells (iPSCs) from somatic cells [51]. iPSCs share with ESCs a genetic signature and many features including pluripotency, differentiative capacity and functional behaviour [52,53,54]. Derivation of iPSCs made it possible to move beyond both the ethical problem of the use of human ESCs and immunorejection, as they can be generated from patient-derived adult cells. Generation of iPSCs is obtained by reprogramming somatic cells to become embryonic-like cells through the introduction of four factors, Oct3/4, Sox2, c-Myc and Klf4 [51]. This process makes it possible to reset the epigenetic and transcriptional profile of adult cells and for each patient to have their own pluripotent stem cells, which can be expanded and differentiated to derive cells or specific tissues. During subsequent years, several methods were developed to reprogram somatic cells by using different delivery vectors to optimise transduction efficiency, each heavily influencing the genetic signature of the derived cells [55,56].

The great potential of cell therapy with iPSCs comes from the possibility of genetically correcting iPSCs obtained from patients with genetic diseases [57,58]. Still, despite the advancements that have been made, limitations persist and include the efficiency of their derivation and the risk of tumour development following transplantation due to the highly proliferative nature of iPSCs and the use of viral vectors for reprogramming [59]. Moreover, the quality of iPSCs is influenced by the adult somatic cells they are derived from, which can accumulate mutations as they age [60] and by the not always well-controlled epigenetic modification that occurs during reprogramming. Taking into account that the choice of the somatic cell type and of the reprogramming method can significantly affect the final product, it is necessary to characterise the biological profile of iPSCs and iPSC-derived cells and better understand the mechanisms underlying reprogramming before using them in a clinical setting. Initial research focused on setting up protocols to differentiate in vitro iPSCs into different precursors or mature cell types lacking the teratogenicity of pluripotent stem cells. This was followed by pre-clinical studies in animal models to test the safety and efficacy of the iPSC-based therapy. The number of clinical trials using iPSCs continues to rise and includes studies on spinal cord injury, macular degeneration, haematological disease, heart disease and neurological diseases [61,62,63]. Autologous cells can only be used as therapy in limited ways due to the time needed to derive iPSCs. This necessarily rules out the use of iPSC-derived cells for acute diseases. To overcome this, some researchers have suggested deriving HLA-typed iPSCs and to bank them for clinical use [64]. However, the long-term engraftment of these cells remains to be demonstrated. Scale-up technologies, including suspension cultures or 3D devices, will help to bring the clinical applicability of iPSCs closer, as long as cell quality is not compromised. Other challenges in the use of iPSC cellular therapeutics include identifying the best harvest site with minimal invasiveness. Moreover, generating autologous iPSC-derived cells for therapy would be very costly and would entail several months for differentiation and quality control.

Adult or somatic stem cells and progenitor cells have been identified in several different tissues and organs. Adult stem cells are multipotent, capable of self-renewal and differentiation into restricted cell types. The main role of adult stem /progenitor cells is to maintain tissue homeostasis throughout life by physiologically renewing cells of the tissues where they reside (cell turnover) and by replacing damaged cells when necessary [42]. Adult stem cells have been isolated from several different tissues including bone marrow, peripheral blood, the brain, skin, skeletal muscle, heart, gut, liver, etc. [42,65,66,67]. As for the kidney, progenitors capable of replacing glomerular cells such as podocytes and tubular epithelial cells, have been identified [68,69,70,71,72,73,74,75,76]. More recently, a new population of perivascular stromal cells has been isolated from the human kidney (kPSCs) [77]. Adult stem cells physically reside in a tissue-specific microenvironment, the niches that provide the necessary support for the maintenance of stemness (self-renewal) and for controlling differentiation. The local niche microenvironment changes throughout life and influences the functions and self-renewal capability of the resident stem cells through different signals [78]. Bone marrow was the first adult tissue to be extensively studied as a potential source of haematopoietic stem cells (HSCs) and mesenchymal stem cells, now named mesenchymal stromal cells (MSCs). HSCs undergo continuous differentiation to produce blood cells to reconstitute the entire haematopoietic system. HSCs are widely used for cell therapy in allogeneic transplantation (Figure 1) [79]. MSCs of adult bone marrow are multipotent stem cells that are capable of self-renewal and differentiation into tissues of mesodermal origin, including bone, cartilage, fat, tendon, muscle and heart [65,80]. Their immunophenotype is characterised by the surface markers CD73, CD90, CD105 and the lack of expression of CD45, CD34, CD14 CD11b, CD79a, CD19 or class II histocompatibility complex antigens [81]. The MSC isolation procedure is simple and based on the capacity of MSCs to adhere to the plastic in vitro, where they exhibit a fibroblast-like morphology. MSCs have been isolated not only from bone marrow but also from the stroma of many other tissues, such as adipose tissue [82], peripheral blood [66], the placenta [83], blood from the umbilical cord [84] and amniotic fluid [85]. MSCs have shown great potential in the field of regenerative medicine, mainly because they are easy to isolate and expand, possess immunosuppressive capacities, do not exhibit any risk of teratoma [86] and are free of ethical concerns. Moreover, in animal studies, MSCs have been shown to be effective therapy in myocardial infarction, kidney diseases, cornea damage, and in lung, brain and spinal cord injuries and in graft versus host disease [87,88,89]. Cell engraftment and the time that cells spend in the target tissues have been evaluated following transplantation because they are important predictors of therapeutic effectiveness. Given the low cell engraftment found in damaged tissues, preconditioning approaches, to increase MSC engraftment and survival rates and efficacy when injected in vivo, have been attempted [90,91]. The cell administration route and the number of injected cells have both been shown to greatly influence cell engraftment and efficacy. The main underlying mechanism through which MSCs contribute to tissue regeneration seems to be the local production of soluble factors that act through endocrine and paracrine pathways [92]. Despite the advantages that MSC-based therapy has, its main limitations are the cells’ poor expansion capacity in vitro and the induction of senescence, which affects cell replication and differentiation capacity. Based on promising experimental studies, the number of clinical trials using MSCs has increased significantly (Figure 1) and raised expectations, as discussed later in this chapter (Table 1).

## 4. Pluripotent Stem Cell Therapy in Experimental Acute Kidney Injury

Pluripotent stem cells, ESCs and iPSCs, have been considered as potential means of tissue regeneration in kidney diseases. The first step in using these cells for therapy consists of differentiating them into renal precursor cells or more mature renal cells. This step is important for both reducing the tumorigenicity intrinsic in pluripotent stem cells and for obtaining cell types that are more prone to differentiating into the desired target tissue. In recent years, several reports have provided protocols for obtaining renal precursors in vitro through the direct conversion or multistep differentiation of iPSCs and ESCs [93,94,95,96,97,98,99]. The use of growth factors and chemical substances has enabled the derivation, with variable efficiency, of populations of nephron precursors that are mainly heterogeneous [95,97,99]. Other differentiation methods used direct reprogramming towards renal cells through transfection vectors (viral or plasmid) [93,100] or synthetic mRNA vectors [101] to force the expression of transcription factors that drive commitment towards renal progenitor cells. The derivation of renal precursor cells from pluripotent stem cells is very useful for multiple purposes, including understanding kidney development, the mechanisms of kidney diseases, the in vitro derivation of kidney disease models, and last but not least, for therapeutic use.

Few reports have focused on the regenerative potential of pluripotent stem cells in kidney diseases, mainly in AKI [96,102,103,104] (Table 2). Our group studied the functional activity of renal progenitor cells (RPCs) derived from human iPSCs in a model of AKI induced by the nephrotoxic drug cisplatin, which is used in the clinic as an anti-tumour agent. RPCs were obtained from iPSCs through a multistep inductive protocol with a nephrogenic cocktail of growth factors [96]. We showed that iPSC-derived RPCs, intravenously injected into mice with AKI, improved renal function and histology. The renoprotective effect of renal precursors was mainly mediated by the ability of these cells to largely engraft the kidney and integrate into damaged tubuli. In parallel, it can be hypothesised that there is a paracrine effect, given the increased proliferation of resident tubular cells that was observed. Later, another study described the efficacy of OSR1^+^SIX2^+^ renal progenitors derived from iPSCs and transplanted under the kidney capsule to improve renal function in terms of reducing blood urea nitrogen (BUN) and attenuating histopathological changes in an AKI model induced by ischaemia and reperfusion (I/R) [102,103]. In both studies, the therapeutic effect of transplanted renal progenitors was mainly due to paracrine effects. Other researchers have reported that iPSC-derived renal precursors encapsulated in hydrogel and injected into the renal parenchyma of rats with I/R had a therapeutic effect on renal function and tubular injury [104] Different cell types have been obtained from iPSC differentiation, including MSC [105] and endothelial cells [106], and have been shown to be effective in AKI models. Notably, iPSC-derived MSCs have been reported to secrete extracellular vesicles (EVs)—membrane-contained vesicles generated by the endoplasmic compartment and plasma membranes [107,108]—which, by incorporating into renal cells, inhibited programmed cell death and finally protected against I/R injury [109].

Whether iPSC-derived renal progenitors could effectively be valuable for treating renal diseases needs to be investigated further.

## 5. Pluripotent Stem cell Therapy in Experimental Chronic Kidney Disease

The therapeutic potential of using pluripotent stem cells to treat CKD has been scarcely addressed yet (Table 2). One study reported that ESCs, transplanted adjacent to injured kidneys rats with 5/6 nephrectomy, slowed the progression of the disease due to the release of paracrine factors [110]. In the same model, another report highlighted that undifferentiated iPSCs injected into the renal parenchyma improved renal damage but caused Wilms’ tumors [111]. MSCs generated from iPSCs when injected into mice with adriamycin (ADR)-induced nephropathy protected against impairment of renal function and had anti-apoptotic effects on tubular cells and anti-fibrotic activities [112]. Unpublished findings from our group showed that iPSC-derived RPCs, which had previously been shown to be effective in the cisplatin-induced AKI model [96], were renoprotective in a model of ADR nephropathy when cells were repeatedly injected. Although iPSC-derived RPCs did not ameliorate proteinuria or glomerulosclerosis, at 14 d after ADR administration, the treatment with iPSC-derived RPCs limited podocyte loss, reduced the number of glomeruli affected by synechiae and reduced vascular rarefaction. Even though these cells exhibited a renal progenitor phenotype, we did not observe renal engraftment or any direct incorporation into glomerular and tubular structures, suggesting that the positive effects of iPSC-derived RPCs were due to a paracrine effect they had on renal tissues. These initial studies suggest it is necessary to potentiate the effect of iPSC-derived cell therapy to obtain clinically relevant outcomes. This could be achieved by identifying iPSC-derived cells with the phenotype of glomerular precursor cells that could be tested in several CKD models to study long-term efficacy and stability.

Ongoing research focuses on understanding whether iPSCs, obtained from patients with chronic kidney diseases, could be a suitable tool for deriving renal precursors that could efficiently regenerate their own kidneys [141,142].

## 6. Adult Stem Cell Therapy in Experimental Acute Kidney Injury

**Figure 2:**
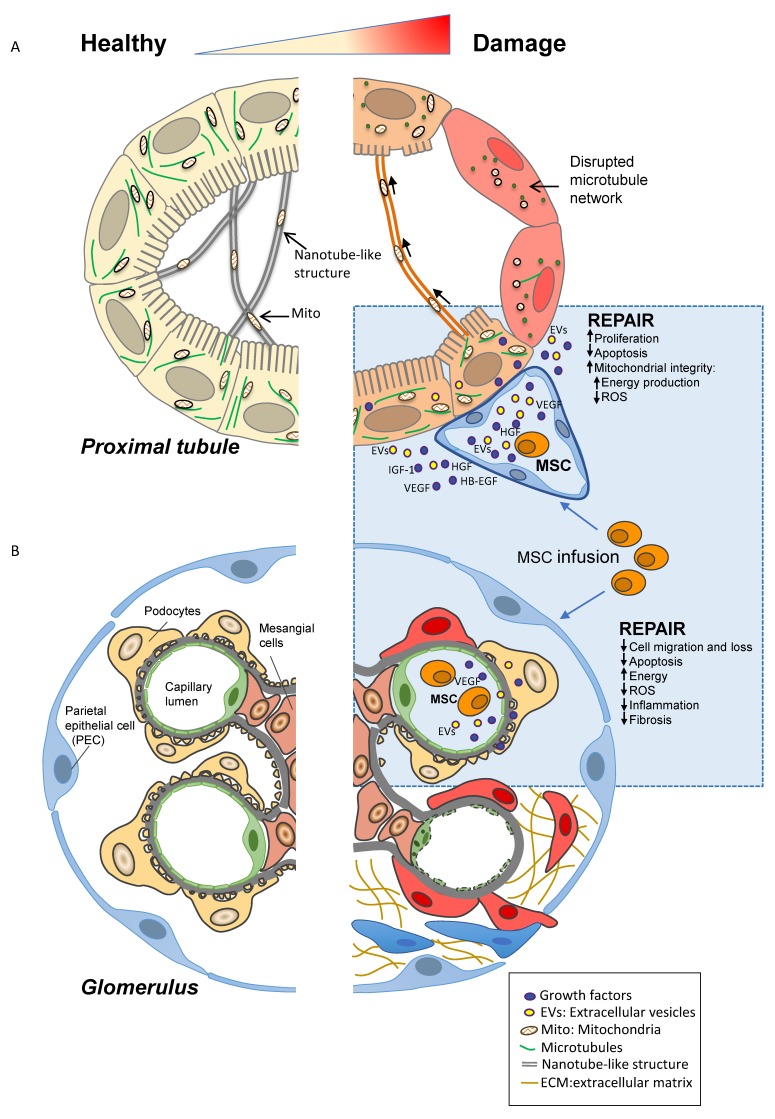
Mechanism of action of MSC-based therapy in kidney diseases. Graphic representation depicting the mechanism of action of MSC-based therapy in (**A**) acute kidney injury and (**B**) chronic kidney disease. (**A**) Following injection, MSCs engraft the damaged kidney and have a protective effect on proximal tubular cells through the local release of growth factors and extracellular vesicles (EVs) with mitogenic, anti-inflammatory and anti-apoptotic effects. Moreover, MSC bioproducts also reduce oxidative stress, sustaining energy supply and mitochondrial exchange among adjacent tubular cells, thus inducing regenerative processes. (**B**) In CKD, upon infusion, MSCs localise in the damaged kidney and limit podocyte migration and loss, glomerular endothelial cell damage and parietal epithelial cell (PEC) activation through the release of growth factors and EVs. MSC therapy by limiting glomerular cell dysfunction also reduces the formation of glomerular fibrotic and sclerotic lesions.

Over the past decade, several different populations of adult stem/progenitor cells have been tested to identify the most promising for stimulating the regenerative ability of the kidney [143] (Table 2). MSCs have been described as one of the most efficient cell populations for activating the regeneration of the damaged kidney (Figure 2A). In this context, the therapeutic effect of MSCs has been demonstrated in different pre-clinical models of AKI [113,114,115,116,117,118]. Our group was the first to observe the renoprotective effects of murine bone marrow (bm) derived MSCs in a murine model of cisplatin-induced AKI [113]. The intravenous injection of murine bmMSCs improved renal function and tubular damage in cisplatin mice, due to their tropism for the damaged kidney [113]. Initial observations attributed the renoprotective effect of MSCs to their ability to engraft at the site of injury and transdifferentiate into renal parenchymal cells [114]. Subsequently, further studies, including our own, revealed that renal MSC engraftment was scarce, transient and cells predominantly localised to the peritubular area of the kidney, suggesting that the main effects of MSCs were probably mediated by paracrine mechanisms on endogenous renal cells [115,116,117]. Actually, in an experimental model of I/R injury, Togel and co-workers provided clear evidence that the administration of bmMSCs had a renoprotective effect through the local secretion of several biologically active factors with anti-apoptotic, immunomodulatory, anti-oxidative and pro-angiogenic properties [117]. Consistent with these findings, our group has identified MSC-derived insulin like growth factors 1 (IGF-1) as a key mediator that is responsible for renal regeneration, as demonstrated by the inability of MSCs silenced for IGF-1 to have a protective effect, either in vivo and in vitro, on cisplatin-induced tubular injury [115]. Using a similar experimental approach, vascular endothelial growth factor (VEGF) produced by MSCs was identified as another important factor that mediates renoprotective effects [116]. In addition, the anti-inflammatory effect of MSCs was described in a model of glycerol-induced AKI where bmMSCs limited inflammation-related injury through the phenotypic switch of macrophages from the pro-inflammatory (M1) to anti-inflammatory (M2) phenotype [118].

With a view to using MSCs in a clinical setting, our group demonstrated that an infusion of human bmMSCs ameliorated renal function and reduced mortality in immunodeficient (NOD–SCID) mice with cisplatin-induced AKI by increasing tubular cell proliferation, preserving microvascular integrity and limiting renal cell apoptosis, in spite of a limited number of engrafted human bmMSCs [119] (Table 2). Bone marrow was the first tissue to be described as being a rich source of MSCs. However, harvesting bone marrow is an invasive procedure and the quantity, frequency, differentiation potential, and maximal life span of MSCs decline with the donor’s age. It has therefore become necessary to search for alternative and more accessible sources of MSCs. Several groups have therefore tested the renoprotective effect of human MSCs isolated from adipose tissue (ad), the umbilical cord (uc) and amniotic fluid (af) in different pre-clinical models of AKI [144] (Table 2). Several studies have described the therapeutic effect of adMSCs in different models of AKI [127,128,145]. Kim et al. have demonstrated that adMSCs reduced inflammation-related molecules and p53, JNK and ERK activation, thus increasing survival time in animals with cisplatin-induced AKI [127]. Another study demonstrated that human adMSCs cultured in low serum (LASCs) secreted higher levels of growth factors such as HGF and VEGF than high serum-cultured human adMSCs (HASCs). When transplanted under the renal capsule of rats with AKI induced by folic acid, LASCs attenuated renal damage and interstitial fibrosis to a greater extent than HASCs [128]. Conversely, intravenous injection of LASCs did not ameliorate AKI [128]. A growing body of evidence has demonstrated the superior therapeutic effect that human MSCs isolated from the umbilical cord have. We found that ucMSCs were renoprotective when systemically injected into mice with AKI and induced tubular cell proliferation and a local inhibitory effect on tubular cell apoptosis, thus prolonging animal survival [132]. Moreover, due to their paracrine effects, ucMSCs were able to limit oxidative stress in response to cisplatin-induced damage and to activate the pro-survival factor Akt, which protected tubular cells from apoptosis [132]. That human ucMSCs create a pro-regenerative environment was also observed in in vitro co-culture where the addition of human ucMSCs to cisplatin-damaged proximal tubular cells enhanced the release of mitogenic and pro-survival factors such as FGF, HB-EGF, VEGF and HGF, while it inhibited the inflammatory cytokines IL-1β and TNFα [132]. Recently, we documented a novel mechanism through which human ucMSCs favour renal repair in cisplatin-induced AKI mice by inducing global metabolic reprogramming of injured tubular cells to sustain energy supply [133]. Specifically, ucMSC-based therapy preserved mitochondrial mass and function by regulating microtubule-rich projections that sustained mitochondrial exchange among adjacent tubular cells [133] (Figure 2A). Furthermore, treatment with ucMSCs stimulated, in injured proximal tubuli, mitochondrial biogenesis, antioxidant defences and energy production through a Sirtuin 3 (SIRT3)-dependent mechanism [133]. Consistent with this, Fang et al. demonstrated that injecting human ucMSCs early after disease induction promoted renal tubular cell proliferation and reduced apoptosis through the modulation of the mitochondrial pathway in folic acid-induced AKI [134].

More recently, an enriched population of mesenchymal stromal cells isolated from human amniotic fluid (hAFS) drew attention due to its greater therapeutic potential, non-tumorigenicity and ethical origins compared to embryonic stem cells [146]. In this context, the therapeutic effect of c-kit positive hAFS cells, which expressed embryonic stem cell markers (Oct-4 and SSEA-4) and mesenchymal stromal markers (CD90, CD105 and CD73) was tested in AKI [137,138]. Despite the potential plasticity of hAFS cells, these cells did not acquire a tubular epithelial phenotype when injected into mice with AKI and exerted their renoprotective effect through the local release of factors such as IL-6, VEGF, and stromal cell-derived factor-1 (SDF-1) [137].

All these pre-clinical studies have underlined how safely and efficiently MSC therapy promotes renal regeneration through complex paracrine activity [144,147] (Figure 2A). However, more research is still needed to overcome the gaps in our knowledge regarding the processes that take place during kidney injury/repair and that impede clinical translation. Moreover, depending on their origin, MSCs have different transcriptomic profiles and differentiation potential and secrete different bioproducts, which can greatly influence their therapeutic efficacy in different diseases in a specific manner. Therefore, the choice of optimal MSC type for the repair of a specific injured renal cell population is a critical issue that must be addressed [148]. Furthermore, the biggest challenge for the applicability of MSC therapies is enhancing their low engraftment in acute but also in chronic kidney injury. To this end, we need to better understand the mechanisms that are at the basis of MSC recruitment, tethering, activation and migration within injured tissues. Then engraftment could be improved by increasing the expression of recruitment molecules through in vitro cell preconditioning strategies, genetic modification to overexpress homing factors, or through cell surface engineering [149].

A number of studies have highlighted the potential that several renal precursor cell populations obtained from rodent and human kidneys and biological fluids, have in the therapy of renal diseases, possibly by directly replacing injured cells [76,150,151,152]. Of these, human kidney-specific progenitor cells isolated from proximal tubules (CD133^+^, CD24^+^, CD106^−^) or from the urinary pole of Bowman’s capsule (CD133^+^, CD24^+^, CD106^+^) were described as exerting a renoprotective effect in a glycerol-induced-AKI model due to their ability to counteract apoptotic stimuli and to directly generate novel tubular cells [75]. Moreover, a new population of perivascular stromal cells has been isolated from the human kidney (kPSCs) [77]. This cell population exhibited a transcriptional profile and stromal cell markers that are similar to human bone marrow cells but also exhibited a renal tissue-specific expression signature including HoxD10 and HoxD11 transcription factors, which are known to be crucial for nephrogenesis [77]. Human kPSCs ameliorated kidney injury when injected into mice with glycerol-induced AKI without incorporating into tubular structures [77]. In spite of the success of renal progenitor cell treatments in experimental kidney diseases, their limited sources, their short life span, and their finite nephrogenic potential limit the applicability of this approach.

Another interesting cell population, endothelial progenitor cells (EPCs) derived from bone marrow or peripheral blood have been studied in different models of toxic or ischaemic-induced AKI demonstrating that they play a major role in maintaining vascular integrity and repairing endothelial damage [139,140,143]. EPC treatment was found to be beneficial through both direct engraftment into injured vascular compartments, where they differentiated into mature endothelium, and also through paracrine mechanisms that resulted in pro-angiogenic effects on resident cells [153].

## 7. Adult Cell-Based Therapy in Experimental Chronic Kidney Disease

A recent systematic review of over 70 articles highlighted MSCs as one of the most effective cell populations for treating experimental CKD [87] (Table 2). Consistently with these observations, our group studied the effects of repeated injections of rat bmMSCs in animals with ADR-induced nephropathy, a model characterised by glomerular podocyte migration and loss and microvessel rarefaction, followed by synechiae formation and glomerular fibrotic lesions [120]. Infusions of bmMSCs significantly normalised glomerular structural alterations, thereby reducing glomerulosclerosis. Nevertheless, bmMSC therapy failed to ameliorate proteinuria in ADR-rats, probably due to the limited recovery of the podocyte slit diaphragm proteins, which were unable to re-establish normal function of foot processes [120]. In the same experimental model, we recently compared the renoprotective effect of human stromal cells of renal origin (kPSCs) to the effect that human stromal cells of non-renal origin (bmMSCs and ucMSCs) have [121]. All three stromal cell populations limited glomerular podocyte loss and endothelial cell injury, attenuating the activation and proliferation of parietal epithelial cells (PECs) (Figure 2B). This translated into a reduction of fibrosis and glomerulosclerosis [121]. Furthermore, we observed that human ucMSCs had an anti-inflammatory effect, which was superior to that of the other stromal cells, reducing macrophage infiltration and inducing the polarisation of pro-inflammatory M1 macrophages towards anti-inflammatory M2 macrophages [121]. Another study, in mice lacking the alpha3-chain of type IV collagen (COL4A3), a model of Alport disease, showed that weekly injections with bmMSCs limited interstitial fibrosis and the loss of peritubular capillaries but failed to ameliorate proteinuria and animal survival [122]. Moreover, bmMSC treatment significantly attenuated renal fibrosis and tubulointerstitial infiltration of macrophages in a murine model of unilateral ureteral obstruction (UUO) [123]. A paracrine activity of human ucMSCs infusion was also demonstrated in a model of CKD in which the amelioration of renal damage was accompanied by significant up-regulation at the renal level of interleukin (IL)-10, heme oxygenase (HO)-1 and HGF expression [135]. In addition, a renoprotective effect of adMSCs on renal fibrosis and inflammation was demonstrated in different models of CKD [129,130,131].

The efficacy of MSC therapy was also investigated in experimental diabetic nephropathy (DN) [124,125]. In a model of DN induced by a high-fat diet and streptozotocin, bmMSC infusion ameliorated albuminuria and limited the expression of pro-inflammatory cytokines and fibrosis in renal tissue via the release of renal trophic factors [126]. Similarly, ucMSCs injected into streptozotocin-induced diabetic rats prevented renal injury and reduced mesangial area expansion and extracellular matrix deposition without affecting hyperglycaemia [136]. Promising results were also obtained by studying the renoprotective effect of human glomerular progenitor cells (CD133^+^, CD24^+^, PDX^−^) given to mice with ADR-induced nephropathy, which were effective in regenerating podocytes and functionally improved glomerular injury [74].

Although several findings have suggested that MSC-based therapy is beneficial in pre-clinical models of CKD (Table 2), the experimental design of these studies varies a lot in terms of CKD models, cell types and doses, administration route, as well as renal outcome parameters, making it difficult to gauge the real therapeutic potential of MSCs and to apply to patients. In this context, a systematic review and meta-analysis, taking into account cell- and CKD model-related aspects, highlighted that MSC-based therapy efficiently improved renal function and morphology and that the route of administration (intravenous or renal artery injection) is a significant predictor of therapeutic efficacy [87]. Surprisingly, the intervention timing in relation to disease onset and the cell dose did not correlate with cell efficacy [87]. This in-depth analysis can be helpful for improving pre-clinical research and supporting the development of clinical trials in the future.

## 8. Therapies with Stem Cell-Derived Bioproducts

As described above, a number of studies have highlighted the importance of the bioactive molecules secreted by stem cells, including MSCs [154,155]. Secretome is defined as a set of factors released by the cells in the extracellular space, such as soluble proteins, free nucleic acids, lipids and extracellular vesicles (EVs) [155,156]. The use of the cell-derived bioproducts instead of cells could have multiple advantages in clinical application, resolving a series of safety concerns that include tumorigenicity, emboli formation and the transmission of infections through injected cells and, last but not least, an easier production and storage process [156]. In this context, cell-free therapy is currently being studied in several pre-clinical models [154,155,156,157]. Convincing evidence indicates that conditioned medium obtained from ucMSCs exerts renoprotective effects similar to those observed with parental cells in an experimental model of cisplatin-induced AKI [133]. Likewise, in a model of ADR-induced nephropathy, ucMSC-derived conditioned medium (CM) limited podocyte loss and glomerular endothelial cell injury, exerting a therapeutic effect similar to that observed with ucMSCs [121]. Other groups have demonstrated that CM obtained from bmMSCs [158] or from ucMSCs reduced renal inflammation and fibrosis and limited the activation of fibrotic signals in a model of unilateral ureteral obstruction (UUO) [159,160]. Moreover, CM derived from bm-derived cells restored microvascular density and decreased the extent of fibrosis in uremic rats with 5/6 nephrectomy by inducing endogenous cell repair [161]. In experimental models of type 1 and type 2 diabetes, treatment with bmMSCs or the corresponding CM also promoted the regeneration of injured kidney tissue, suppressing cell infiltration and reducing interstitial fibrosis and glomerular cell alterations to a comparable extent [126].

In addition to soluble factors, EVs such as exosomes (Exs, 30–100 nm diameter) and microvesicles (MVs, 100–1000 nm diameter) have been described as a different form of cell-to-cell communication through the horizontal transfer of microRNAs (miRNAs), mRNAs, and proteins to target cells [107,108]. Several studies have shown that treatment with MSC-derived EVs is safe and has renoprotective effects in models of AKI and CKD [155,157]. In mice with glycerol-induced AKI, the infusion of human bmMSC-derived EVs had a beneficial renal effect [162] through the transfer of MSC-specific subsets of mRNAs and miRNAs to tubular cells, regulating proliferation, differentiation, cell survival and immunoregulation [163,164,165]. Likewise, the renoprotective effect of EVs derived from human ucMSC was demonstrated in different experimental models of AKI [166,167,168]. Among these studies, in a model of cisplatin-induced AKI, ucMSCs-Exs reduced tubular cell apoptosis and oxidative stress [169]. In addition, MVs have also been described to transfer HGF mRNA from ucMSCs to tubular cells, thus increasing their dedifferentiation and growth and activating regenerative mechanisms in IRI [170]. In this context, our group demonstrated that bone marrow MSC-derived EVs were responsible for the horizontal transfer of IGF-1 receptor mRNA to tubular cells damaged by cisplatin [171]. Bone marrow-derived MSCs shuttling this genetic information, together with the secretion of IGF-1, rendered tubular cells more inclined to the regeneration triggered by stem cell therapy.

A growing body of evidence indicates that the administration of EVs derived from MSCs of various origins also improved renal function and renal structural recovery in different CKD models, including metabolic syndrome and renal artery stenosis, UOO, and DN [172,173,174]. The renoprotective effect of MSCs can be mediated by secreted factors and EVs containing mRNA, miRNA or proteins that, working in synergy, can induce renal protection and regeneration after injury, as summarised in Figure 2.

Overall, the translation of MSC-derived extracellular vesicles, more specifically exosomes, into a therapeutically valuable agent, presents several challenges, including the optimisation of their isolation and characterisation due to the absence of specific biomarkers. The lack of a method for evaluating the complexity of the exosome cargo and the possible interference of unknown secreted factors raise concerns regarding its future application. Nevertheless, regulatory requirements for large-scale manufacturing and quality control remain ill defined.

## 9. Stem Cell Therapies in Clinical Trials

As can be seen in the present, up-to-date review, numerous studies highlight the progress and advances that have been made with stem cell products as a novel therapy for various diseases. The potential of different populations of stem/progenitor cells to limit structural and functional damage and to stimulate the regenerative capacity of the injured tissues and/or organs has been described extensively in pre-clinical models. Nonetheless, in this novel field that holds great promise, differences in methodologies and contradictory results have delayed clinical translatability for patients.

In general, based on their therapeutic potential, MSCs from bone marrow or other tissues are considered one the most powerful tools for treating several human diseases. A number of clinical trials have been designed to evaluate the safety and efficacy of MSC-based therapy and to highlight the main criticisms of the use of these cell populations in different disorders (Figure 1). To date (08/04/2019), over 880 MSC-based clinical trials, complete or ongoing, have been registered in the US National Institutes of Health (www.clinicaltrial.gov) database, including haematological diseases, graft versus host disease, diabetes, organ transplantation, inflammatory diseases, and diseases in the lung, liver, bone as well as cardiovascular, neurological and autoimmune diseases [175,176,177,178] (Figure 1). The majority of the above clinical trials are carried out in early phases (phase I–II), suggesting that the efficiency of MSC treatment remains to be further investigated in the long-term. This consideration is particularly true for kidney-related diseases, where there have been over 30 phase I/II clinical trials that focused on the safety and feasibility of allogeneic or autologous administration of human MSCs from bone marrow, adipose tissue or umbilical cord origin, spanning a wide range of diseases that have been summarised briefly below [177,178] (Figure 1, Table 1).

Based on a large body of pre-clinical evidence regarding the effectiveness of MSCs in AKI, a number of studies were proposed to test the safety and efficacy of allogeneic bmMSC infusions, in patients at high risk of developing AKI. A recently completed phase I exploratory study (NTC00733876), demonstrated the feasibility and efficacy of escalating doses of bmMSCs infused in the suprarenal aorta in 16 patients undergoing on-pump cardiac surgery [9,10]. Infusions of MSCs were safe and protected against early and late post-surgery renal function deterioration, thus decreasing hospital stays and readmission rates compared to historical case controls [9,10]. These promising results were not supported by a randomised double-blind phase II study (NTC01602328) in a similar cohort of 156 patients with established AKI 48h after surgery, who received intra-aortic administration of allogeneic MSCs (AC607 2 × 10^6^ cells/kg, Allocure) or placebo. Although treatment with MSCs was found to be safe and tolerated well, no protective effect on renal function and mortality was observed [11].

As for chronic non-proteinuric nephropathy, six patients with autosomal dominant polycystic kidney disease received autologous bmMSCs (1–2 × 10^6^ cells/kg) to evaluate their safety and tolerability (NCT02166489). No cell-related adverse effects occurred over the 12-month follow-up, although no amelioration was detected in renal function and kidney length, considered as a surrogate index of renal growth [12]. This negative result may be ascribable to the small number of patients and to the advanced stage of the disease they were at when they received cell therapy. In this context, perhaps the use of MSCs derived from patients with a genetic disorder should be also considered.

Recently, based on a first report [13] that demonstrated, in a paediatric patient with focal segmental glomerulosclerosis (FSGS), the absence of adverse effects of three cycles of two infusions of allogeneic bmMSCs (1 × 10^6^ cells/kg), a clinical trial was subsequently begun and is ongoing. Its aim is to evaluate the safety and efficacy of the intravenous administration of allogeneic adMSCs in five FSGS patients who have not responded to conventional or unconventional treatments.

As for type II diabetes, two randomised clinical trials have recently been published [179]. In the first study, 61 enrolled diabetic patients with no renal involvement received between one and three doses of MSCs (rexlemestrocel-L therapy: 0.3, 1.0, 2 × 10^6^ cells/kg) [179]. Cell transplantation was found to be safe and glycated haemoglobin was found to be lower (7%) in 33% of patients over 12 weeks of follow-up. The second trial enrolled patients with DN who received a single, higher dose of rexlemestrocel-L (150 or 300 × 10^6^ MSCs) or placebo and were followed up for 60 weeks [14]. Cell therapy was safe and tolerated well but no reduction of glycemic levels was observed in this group of DN patients. An interesting observation in this study was the trend towards stabilisation of eGFR that occurred in patients over the 60-week follow-up [14]. More recently, a controlled phase I/II clinical trial (NTC02585622) began to determine the safety of therapy with allogenic bmMSCs (ORBCEL-MN, 80, 160 and 240 × 10^6^ cells) with patients with progressive DN with mild to moderate renal insufficiency. This multinational study is valuable due to the use of a well-characterised MSC population and the longer period of observation that has followed.

Another promising application of MSC-based therapy includes atherosclerotic renovascular hypertension as shown in an explorative phase I/II study (NCT02266394) that was recently completed. It demonstrated increased cortical perfusion and renal blood flow with the stabilisation of measured GFR at 3 month follow-up in 14 patients who were treated with escalating doses of autologous adMSCs (1 or 2.5 × 10^5^ cells/kg) [15]. A subsequent phase I study that is ongoing was designed to test whether the infusion of autologous adMSCs—in patients with large vessel renovascular disease before percutaneous transluminal angioplasty with stenting—can enhance renal blood flow and ameliorate renal function.

The therapeutic benefit of MSCs has also been investigated in autoimmune diseases such as systemic lupus erythematosus (SLE). Three phase I/II clinical trials examined the effects of allogeneic bmMSC or ucMSC infusions in patients with primary and refractory SLE [16,17,18]. Following an initial study (NTC00698191) with four patients [16], the same group demonstrated in a larger group of patients that infusion of allogeneic bmMSCs (1 × 10^6^ cells/kg) improved the SLE disease activity index score and decreased circulating autoreactive antibodies, with a reduction of proteinuria [17]. Similar results were obtained with ucMSC-based therapy. These studies reported no adverse effects related to cell treatments at one year of follow-up and also described complete or partial remission in 30–50% of patients with SLE. Conversely, a recent multicenter randomised, double-blind controlled trial with eighteen SLE patients treated with ucMSCs did not demonstrate that there was any protective effect. The lack of correct cell characterisation impedes proper analysis of the study [19].

Given the immunomodulatory properties of MSCs that tip the balance towards T regulatory cell expansion, MSC-based therapy has been studied in patients who received kidney transplants, with the aim of controlling the host immune-response towards the graft and minimising immunosuppression, possibly leading to transplant tolerance [178,180]. To date, the results from six phase I clinical studies are available and, of these, four studies were performed using autologous MSCs [20,21,22,23,24], while the other two used allogeneic MSCs [25,26]. Twelve other studies are ongoing and no data have been provided yet. Notably, the available data on kidney transplantation have clearly shown that infusions of bmMSCs (dose ranging between 1–2 × 10^6^ /kg body weight) are safe, tolerated well and do not have significant side effects in the short or long term if combined with an adequate immunosuppressive regimen. Immunomonitoring these patients revealed increased circulating CD4^+^ CD25^+^ FOXP3^+^ Tregs, a reduction in the number of memory CD8^+^ Tcells, and long lasting donor-specific hyporesponsiveness of CD8^+^ T cells in ex vivo tests [181]. The generation of the B cell signature has been accompanied by immunotolerance. These preliminary data suggest that, if combined with the appropriate immunosuppressive regimen, MSCs in kidney transplants can dampen immune responses and pave the way for well-designed clinical trials to definitively demonstrate the pro-tolerogenic potential of MSCs.

## 10. Conclusions

The promise of stem cell therapies in pre-clinical models of kidney diseases is yet to be translated into more persuasive proof of clinical efficacy. Several clinical trials have confirmed the safety and tolerability of stem cells, and in particular of MSC-based therapies, in patients with renal diseases and kidney transplants (Table 1). However, long-term monitoring is recommended to rule out the potential risk of cancer and of developing anti-HLA antibodies. Furthermore, the heterogeneity of MSCs obtained from different tissues and the lack of standardised protocols for isolation and in vitro culture are potential confounding variables that make the comparison of different clinical trials difficult, as highlighted recently [148,178,182]. The challenge that remains is to better understand the heterogeneous landscape of different stem cell populations and their secretomes so that the criteria for designing clinical studies with high impact can be defined. Furthermore, the clinical implementation of these innovative strategies is limited by a lack of knowledge regarding the in vivo mechanisms of action of pluripotent and adult stem cell-based therapies. A greater understanding of their behaviour would make it possible to identify the optimal cellular source, best administration route and correct dosing for different kidney diseases, as well as the specific biomarkers that should be observed to monitor treatment responses.

## Figures and Tables

**Table 1:** Clinical trials with human MSCs in kidney diseases.

Studies	No. of Patients (Follow-Up)	MSC (Source, Dose, and Timing)	Main Results	References
**AKI**				
Togel and Westernfelder and Gooch et al. NCT00733876	16 patients undergoing on-pump cardiac surgery at high risk of postoperative AKI; (16 mo)	Allogeneic bmMSCs (Allocure) escalating doses (not specified), i.a. infusions	Cells were safe and protected against early and late post-surgery renal function deterioration, decreased length of hospital stay and need for readmission	[9,10]
Swaminathan et al. NCT01602328	156 patients with established AKI 48h after cardiac surgery; n = 77 treated with MSCs, n = 79 placebo, (90 d)	Allogeneic bmMSCs (Allocure), single i.a. infusion of 2 × 10^6^ cells/kg	MSC infusion was safe and well tolerated. No difference in time to recovery of kidney function, dialysis and mortality compared with placebo	[11]
**CKD**				
Makhlough et al. NCT02166489	6 patients with autosomal dominant polycystic kidney disease (ADPKD); (12 mo)	Autologous bmMSCs, single i.v. injection of 1–2 × 10^6^ cells/kg	MSC infusion was safe and well tolerated. No significant changes in eGFR, SCr or kidney length	[12]
Belingheri et al.	1 paediatric patient with recurrent focal segmental glomerulosclerosis (FSGS) after renal transplantation; (22 mo)	Allogeneic bmMSCs, i.v. injection of 1 × 10^6^ cells/kg/dose for 6 doses	MSC infusion was safe and well tolerated. and maintained stable uPr/uCr ratio	[13]
Packham DK et al. NCT01843387	30 patients with diabetic nephropathy (DN); n = 10 lower bmMPC dose, n = 10 higher bmMPC dose, n = 10 placebo; (60 wk)	Allogeneic bmMPCs, single i.v. injection of lower dose (150 × 10^6^ cells/patient) or higher dose (300 × 10^6^ cells/patient)	bmMPC infusion was safe and well tolerated. Stabilization and improvement of eGFR in bmMPC 150 × 10^6^ group	[14]
Saad et al. NCT01840540	28 patients with atherosclerotic renal vascular disease; n = 7 lower adMSC dose, n = 7 higher adMSC dose, n = 14 placebo; (3 mo)	Autologous adMSCs, single i.a. infusion of lower dose (1 × 10^5^ cells/kg) or higher dose (2.5 × 10^5^ cells/kg)	MSC infusion was safe and well tolerated. Increase in cortical perfusion and renal blood flow and stabilization of GFR	[15]
**Lupus Nephritis**				
Sun et al. and Liang et al. NTC00698191	13 patients with refractory SLE; ( > 12 mo)	Allogeneic bmMSCs, single i.v. injection of 1 × 10^6^ cells/kg	MSC infusion was safe and well tolerated. Amelioration in SLEDAI score and proteinuria. (Two patients had a relapse of proteinuria)	[16,17]
Sun et al. NTC00698191	16 patients with refractory SLE; (8 mo)	ucMSCs, single i.v. injection of 1 × 10^6^ cells/kg	MSC infusion was safe and well tolerated. Improvement in SLEDAI score and renal function	[18]
Deng et al. NTC01539902	18 patients with lupus nephritis; n = 12 ucMSCs, n=6 placebo; (12 mo)	ucMSCs, two i.v. injections of 2 × 10^8^ cells/patient, 7d apart	MSC infusion was safe and well tolerated. No effect of ucMSCs above standard immunosuppression	[19]
**Kidney trasplantation**				
Perico et al. NTC00752479	2 living donor kidney Tx recipients; (1 yr)	Autologous bmMSCs, single i.v. infusion of 1.7–2 × 10^6^ cells/kg, day +7 post-Tx	MSC infusion was safe and well tolerated. Transient enhancement of serum creatinine levels after MSC infusion. Increased of the percentage of Treg and inhibition of memory CD8^+^ T cell expansion	[20]
Perico et al. NTC02012153	2 living donor kidney Tx recipients; (1 yr)	Autologous bmMSCs, single i.v. infusion of 2 × 10^6^ cells/kg, day −1 pre-Tx	MSC infusion was safe and well tolerated. No MSC-associated renal insufficiency. Decrease in circulating memory CD8^+^ T and donor-specific CD8^+^ T cell cytolitic response	[21]
Tan et al. NTC00658073	159 living donor kidney Tx recipients; n = 53 bmMSCs + std. dose CNI; n = 52 bmMSCs + low dose CNI (80% of std); n = 51 basiliximab + std. CNI; (1 yr)	Autologous bmMSCs, two i.v. infusions of 1–2 × 10^6^ cells/kg, day 0 and day +14 post-Tx	MSC infusion was safe and well tolerated. MSC infusion showed lower incidence of acute rejection, descreased risk of opportunistic infections and had faster renal function recovery compared with controls.	[22]
Reinders et al. NTC00734396	6 living donor kidney Tx recipients; (6 mo)	Autologous bmMSCs, two i.v. injections of 0.1–1 × 10^6^ cells/kg, 7 d apart at 6 mo post-Tx	MSC infusion was safe and well tolerated. Increased incidence of opportunistic infections. Resolution of tubulitis and interstitial fibrosis/tubular atrophy in two patients	[23]
Mudrabettu et al. NTC02409940	4 living donor kidney Tx recipients; (6 mo)	Autologous bmMSCs, two i.v. infusions of 0.2–0.8 × 10^6^ cells/kg, day −1 and day +30 post-Tx	MSC infusion was safe and well tolerated. Increase in regulatory T cells and reduction in CD4^+^ T cell proliferation	[24]
Pan et al.	32 living donor kidney Tx recipients; n = 16 MSC + low dose tacrolimus (50%of std), n = 16 std. tacrolimus dose controls; (2 yr)	Allogeneic bmMSCs, two infusions: 5 × 10^6^ into renal artery at day 0 and 2 × 10^6^ cells/kg at day +30 post-Tx	MSC infusion was safe and well tolerated. Comparable incidence of acute rejection and similar graft function and survival between patient groups. MSCs allow to use a lower dose of tacrolimus	[25]
Erpicum et al. NCT01429038	10 deceased donor kidney Tx recipients; (1 yr)	Allogeneic bmMSCs, single i.v. injections: 1.5 × 10^6^–3 × 10^6^ at day +3 post-Tx	MSC infusion was safe and well tolerated. Increase in regulatory T cells and improvement of early allograft function. Long-term effects and immunization against MSC, remain to be studied.	[26]

AKI: acute kidney injury; mo: month; i.a.: intra-arterial; d: day; yr: year; i.v.: intravenous; bmMSCs: bone marrow-derived mesenchymal stromal cells; CKD: chronic kidney disease; bmMPC: bone marrrow-derived mesenchymal precursor cell; adMSCs: adipose tissue-derived mesenchymal stromal cells; GFR: glomerular filtration rate; eGFR: estimated glomerular filtration rate; SLE: systemic lupus erythematosus; SLEDAI: SLE Disease Activity Index; ucMSCs: umbilical cord-derived mesenchymal stromal cells; Tx: Transplantation; std., standard; CNI: calcineurin inhibitor; SCr: serum creatinine.

**Table 2:** Stem cell-based therapy in experimental kidney diseases.

Donor Cells	References	
	AKI	CKD
**ESCs and iPSC-derived RPCs**	[96,102,103,104]	[110,111,112]
**bmMSCs**	[113,114,115,116,117,118,119]	[120,121,122,123,124,125,126]
**adMSCs**	[127,128]	[129,130,131]
**ucMSCs**	[132,133,134]	[121,135,136]
**AFS cells**	[137,138]	
**RPCs**	[75]	[74]
**kPSCs**	[77]	[121]
**EPCs**	[139,140]	[87]

AKI: acute kidney injury; CKD: chronic kidney disease; iPSCs: induced pluripotent stem cells; ESCs: embryonic stem cells; bmMSCs: bone marrow-derived mesenchymal stromal cells; adMSCs: adipose tissue-derived mesenchymal stromal cells; ucMSCs: umbilical cord-derived mesenchymal stromal cells; AFS: amniotic fluid stem cells; RPCs: renal progenitor cells; kPSCs: kidney perivascular stromal cells; EPCs: endothelial progenitor cells.
